# Crystal structure of 2-(1*H*-imidazol-3-ium-4-yl)ethanaminium dichloride, a re-determination

**DOI:** 10.1107/S2056989015018848

**Published:** 2015-10-14

**Authors:** Samira Louhibi, Imene Belfilali, Leila Boukli-Hacene, Thierry Roisnel

**Affiliations:** a Laboratoire de Chimie Inorganique et Environnement, Universite de Tlemcen, BP119, 13000, Tlemcen, Algeria; b Centre de Diffractometrie X, UMR 6226 CNRS, Unite Sciences Chimiques de Rennes, Universite de Rennes I, 263 Avenue du General Leclerc, 35042 Rennes, France

**Keywords:** crystal structure, histamine, redetermination, hydrogen bonding

## Abstract

The crystal structure of the title mol­ecular salt, C_5_H_11_N_3_^+^·2Cl^−^, was redetermined. In comparison with the previous study [Bonnet *et al.* (1975 ▸). *Bull. Soc. Fr. Mineral. Crist.* **98**, 208–213.], the positions of some H atoms were corrected, allowing a more accurate description of the hydrogen-bonding scheme. In addition, the absolute structure was also determined. The maximum differences in terms of bond lengths and angles between the two determinations are 0.022 Å and 1.43°, respectively. The organic cation display a *anti* conformation of the protonated amine function and the imidazolium ring. The dihedral angle between the imidazolium plane and the plane through the C—C—N side chain is 29.58 (3)°. In the crystal, the organic cations and Cl^−^ anions are stacked alternatively into layers parallel to (100). N—H⋯Cl hydrogen bonds between all H atoms of the ammonium group and both N—H groups of the imidazolium ring and the Cl^−^ acceptor anions lead to the linkage of organic and inorganic layers into a three-dimensional network.

## Related literature   

Histamine [2-(1*H*-imidazol-4-yl)ethanamine] is a biogenic amine present in essentially all mammalian tissues and involved in several defense mechanisms of the body. It plays a role in various physiological processes, such as control of gastric acid secretion, neurotransmission, regulation of the microcirculation, and modulation of inflammatory (Cooper *et al.*, 1990 ▸; Barnes, 2001 ▸) and immunological reactions (Schwartz *et al.*, 1991 ▸; Bachert *et al.*, 1998 ▸; Emanuel *et al.*, 1999 ▸). The contribution of histamine in these physiological and pathological processes and the use in pharmacology make it an inter­esting substance in biochemistry (Leurs *et al.*, 1995 ▸; Galoppin & Ponvert, 1997 ▸; O’Mahony *et al.*, 2011 ▸; Jadidi-Niaragh & Mirshafiey, 2010 ▸; Gustiananda *et al.*, 2012 ▸). The structure of the title compound has been determined previously by Bonnet *et al.* (1975 ▸) who reported lattice parameters of *a* =7.596 (6), *b* = 12.706 (8), *c* = 4.457 (4) Å, β = 91.64 (5)° at room temperature. For the structure of the histamine copper(II) chloride complex and its catalytic activity study, see: Belfilali *et al.* (2015*a* ▸), and for the structure of monopronated histamine with Cl^−^ as counter-anion, see: Belfilali *et al.* (2015*b* ▸).
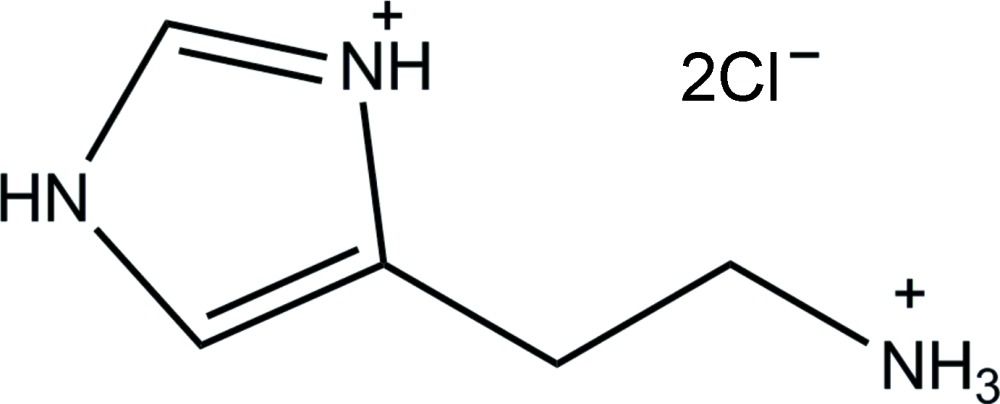


## Experimental   

### Crystal data  

C_5_H_11_N_3_^+^·2Cl^−^
*M* *_r_* = 184.07
Monoclinic, 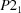

*a* = 4.4358 (2) Å
*b* = 12.6281 (4) Å
*c* = 7.5588 (3) Å
β = 91.910 (1)°
*V* = 423.18 (3) Å^3^
*Z* = 2
Mo *K*α radiation
μ = 0.70 mm^−1^
*T* = 150 K
0.43 × 0.32 × 0.09 mm

### Data collection  

Bruker APEXII CCD, diffractometer
Absorption correction: multi-scan (*SADABS*; Bruker, 2006 ▸) *T*_min_ = 0.825, *T*_max_ = 0.939
3729 measured reflections
1855 independent reflections
1815 reflections with *I* > 2σ(*I*)
*R*_int_ = 0.024

### Refinement  

*R*[*F* ^2^ > 2σ(*F* ^2^)] = 0.023
*wR*(*F* ^2^) = 0.058
*S* = 1.08
1855 reflections
106 parameters
1 restraint
H atoms treated by a mixture of independent and constrained refinement
Δρ_max_ = 0.22 e Å^−3^
Δρ_min_ = −0.17 e Å^−3^
Absolute structure: Flack (1983 ▸), 841 Friedel pairs
Absolute structure parameter: 0.06 (5)

### 

Data collection: *APEX2* (Bruker, 2006 ▸); cell refinement: *SAINT* (Bruker, 2006 ▸); data reduction: *SAINT*; program(s) used to solve structure: *SIR97* (Altomare *et al.*, 1999 ▸); program(s) used to refine structure: *SHELXL97* (Sheldrick, 2008 ▸); molecular graphics: *ORTEP-3 for Windows* (Farrugia, 2012 ▸); software used to prepare material for publication: *WinGX* (Farrugia, 2012 ▸).

## Supplementary Material

Crystal structure: contains datablock(s) I. DOI: 10.1107/S2056989015018848/wm5220sup1.cif

Structure factors: contains datablock(s) I. DOI: 10.1107/S2056989015018848/wm5220Isup2.hkl

Supporting information file. DOI: 10.1107/S2056989015018848/wm5220Isup3.cml

**Fig. 1.**
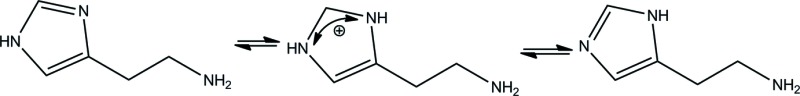
Tautomeric forms of histamine.

. DOI: 10.1107/S2056989015018848/wm5220fig1.tif
Tautomeric forms of histamine.

**Fig. 2.**
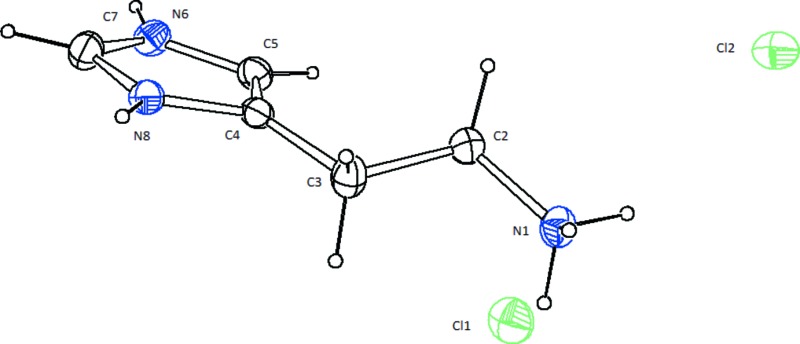
The molecular structure of the title compound. Displacement ellipsoids are drawn at the 50% probability level.

. DOI: 10.1107/S2056989015018848/wm5220fig2.tif
The mol­ecular structure of the title compound. Displacement ellipsoids are drawn at the 50% probability level.

**Fig. 3.**
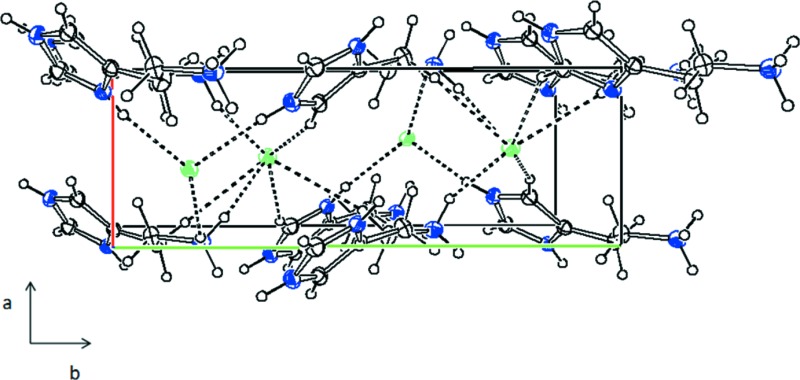
Part of the crystal structure with hydrogen bonds shown as dashed lines.

. DOI: 10.1107/S2056989015018848/wm5220fig3.tif
Part of the crystal structure with hydrogen bonds shown as dashed lines.

CCDC reference: 1051848

Additional supporting information:  crystallographic information; 3D view; checkCIF report

## Figures and Tables

**Table 1:** Hydrogen-bond geometry (, )

*D*H*A*	*D*H	H*A*	*D* *A*	*D*H*A*
N1H1*A*Cl1^i^	0.92(3)	2.19(3)	3.1071(17)	171(2)
N1H1*B*Cl1	0.95(3)	2.34(3)	3.1930(17)	150(2)
N1H1*C*Cl2^i^	0.89(3)	2.44(3)	3.1768(16)	141(2)
N6H6Cl2^ii^	0.88(3)	2.28(3)	3.1046(16)	157(2)
N8H8Cl2^iii^	0.84(3)	2.28(3)	3.1157(15)	169(3)

Symmetry codes: (i) 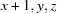; (ii) 
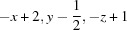
; (iii) 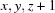.

## supplementary crystallographic information

### S1. Synthesis and crystallization

A stoichiometric mixture of histamine di­hydro­chloride (1.0 mmol) and 3-eth­oxy-4-hy­droxy­benzaldehyde was irradiated in a microwave oven at 200 Watt for 10 minutes. The reaction mixture was then allowed to attain room temperature and the obtained crystals were separated by filtration.

### S2. Refinement

Hydrogen atoms linked to nitro­gen atoms were found from Fourier difference maps and were refined with distance restraints in the range 0.84 (3) - 0.95 (3) Å and with a common *U*_iso_ parameter of 0.05 Å^2^. C-bound hydrogen atoms were refined with calculated positions, with C—H = 0.95 and *U*(H)_iso_ = 1.2*U*_eq_(C) for aromatic H atoms and with C—H = 0.99 and *U*(H)_iso_ = 1.2*U*_eq_(C) for methyl­ene H atoms.

### Crystal data

**Table:**

C_5_H_11_N_3_^+^·2Cl^−^	*F*(000) = 192
*M**_r_* = 184.07	*D*_x_ = 1.445 Mg m^−^^3^
Monoclinic, *P*2_1_	Mo *K*α radiation, λ = 0.71073 Å
Hall symbol: P 2yb	Cell parameters from 2786 reflections
*a* = 4.4358 (2) Å	θ = 2.7–27.5°
*b* = 12.6281 (4) Å	µ = 0.70 mm^−^^1^
*c* = 7.5588 (3) Å	*T* = 150 K
β = 91.910 (1)°	Prism, colourless
*V* = 423.18 (3) Å^3^	0.43 × 0.32 × 0.09 mm
*Z* = 2	

### Data collection

**Table:**

Bruker APEXII CCD, diffractometer	1815 reflections with *I* > 2σ(*I*)
Graphite monochromator	*R*_int_ = 0.024
CCD rotation images, thin slices scans	θ_max_ = 27.5°, θ_min_ = 3.1°
Absorption correction: multi-scan (*SADABS*; Bruker, 2006)	*h* = −5→5
*T*_min_ = 0.825, *T*_max_ = 0.939	*k* = −16→13
3729 measured reflections	*l* = −9→9
1855 independent reflections	

### Refinement

**Table:**

Refinement on *F*^2^	Secondary atom site location: difference Fourier map
Least-squares matrix: full	Hydrogen site location: inferred from neighbouring sites
*R*[*F*^2^ > 2σ(*F*^2^)] = 0.023	H atoms treated by a mixture of independent and constrained refinement
*wR*(*F*^2^) = 0.058	*w* = 1/[σ^2^(*F*_o_^2^) + (0.0327*P*)^2^] where *P* = (*F*_o_^2^ + 2*F*_c_^2^)/3
*S* = 1.08	(Δ/σ)_max_ = 0.001
1855 reflections	Δρ_max_ = 0.22 e Å^−^^3^
106 parameters	Δρ_min_ = −0.17 e Å^−^^3^
1 restraint	Absolute structure: Flack (1983), 841 Friedel pairs
Primary atom site location: structure-invariant direct methods	Absolute structure parameter: 0.06 (5)

### Special details

**Table:**

Geometry. All e.s.d.'s (except the e.s.d. in the dihedral angle between two l.s. planes) are estimated using the full covariance matrix. The cell e.s.d.'s are taken into account individually in the estimation of e.s.d.'s in distances, angles and torsion angles; correlations between e.s.d.'s in cell parameters are only used when they are defined by crystal symmetry. An approximate (isotropic) treatment of cell e.s.d.'s is used for estimating e.s.d.'s involving l.s. planes.
Refinement. Refinement of *F*^2^ against ALL reflections. The weighted *R*-factor *wR* and goodness of fit *S* are based on *F*^2^, conventional *R*-factors *R* are based on *F*, with *F* set to zero for negative *F*^2^. The threshold expression of *F*^2^ > σ(*F*^2^) is used only for calculating *R*-factors(gt) *etc*. and is not relevant to the choice of reflections for refinement. *R*-factors based on *F*^2^ are statistically about twice as large as those based on *F*, and *R*- factors based on ALL data will be even larger.

### Fractional atomic coordinates and isotropic or equivalent isotropic displacement parameters (Å^2^)

**Table:**

	*x*	*y*	*z*	*U*_iso_*/*U*_eq_	
N1	0.9759 (4)	0.18301 (13)	0.4308 (2)	0.0201 (3)	
H1A	1.115 (6)	0.232 (2)	0.472 (3)	0.05*	
H1B	0.787 (6)	0.217 (2)	0.441 (3)	0.05*	
H1C	1.015 (6)	0.179 (2)	0.316 (4)	0.05*	
C2	0.9990 (5)	0.07908 (15)	0.5236 (2)	0.0209 (4)	
H2A	1.2044	0.0497	0.5125	0.025*	
H2B	0.853	0.0285	0.469	0.025*	
C3	0.9317 (4)	0.09457 (15)	0.7190 (2)	0.0214 (4)	
H3A	1.053	0.1545	0.7669	0.026*	
H3B	0.716	0.1127	0.7299	0.026*	
C4	1.0019 (4)	−0.00243 (13)	0.8252 (2)	0.0160 (3)	
C5	1.1983 (4)	−0.08313 (15)	0.8024 (2)	0.0189 (3)	
H5	1.3185	−0.0945	0.7025	0.023*	
N6	1.1904 (3)	−0.14577 (12)	0.95191 (19)	0.0206 (3)	
H6	1.284 (6)	−0.206 (2)	0.964 (3)	0.05*	
C7	0.9952 (4)	−0.10582 (15)	1.0615 (2)	0.0211 (4)	
H7	0.9475	−0.1342	1.1736	0.025*	
N8	0.8764 (3)	−0.01892 (12)	0.98803 (17)	0.0168 (3)	
H8	0.745 (6)	0.021 (2)	1.031 (4)	0.05*	
Cl1	0.47163 (8)	0.32961 (3)	0.59693 (5)	0.02068 (11)	
Cl2	0.43148 (9)	0.15270 (3)	0.11930 (5)	0.01937 (11)	

### Atomic displacement parameters (Å^2^)

**Table:**

	*U*^11^	*U*^22^	*U*^33^	*U*^12^	*U*^13^	*U*^23^
N1	0.0192 (7)	0.0233 (9)	0.0178 (7)	−0.0004 (6)	0.0021 (6)	0.0025 (6)
C2	0.0264 (9)	0.0187 (9)	0.0175 (9)	−0.0022 (7)	0.0010 (7)	−0.0001 (7)
C3	0.0267 (9)	0.0202 (9)	0.0174 (8)	0.0047 (8)	0.0036 (7)	0.0012 (7)
C4	0.0168 (7)	0.0158 (8)	0.0155 (7)	−0.0017 (7)	0.0002 (6)	−0.0027 (6)
C5	0.0208 (8)	0.0177 (8)	0.0183 (8)	0.0006 (7)	0.0026 (6)	−0.0036 (7)
N6	0.0214 (7)	0.0173 (8)	0.0233 (7)	0.0014 (6)	0.0018 (6)	0.0014 (6)
C7	0.0225 (9)	0.0215 (9)	0.0195 (7)	−0.0013 (7)	0.0008 (7)	0.0033 (7)
N8	0.0167 (7)	0.0168 (7)	0.0173 (7)	−0.0009 (6)	0.0032 (5)	−0.0017 (5)
Cl1	0.01948 (19)	0.0235 (2)	0.01936 (17)	−0.00067 (18)	0.00474 (13)	−0.00219 (16)
Cl2	0.01775 (18)	0.0187 (2)	0.02194 (18)	−0.00186 (17)	0.00442 (13)	−0.00354 (15)

### Geometric parameters (Å, º)

**Table:**

N1—C2	1.490 (2)	C4—C5	1.355 (3)
N1—H1A	0.92 (3)	C4—N8	1.383 (2)
N1—H1B	0.95 (3)	C5—N6	1.381 (2)
N1—H1C	0.89 (3)	C5—H5	0.95
C2—C3	1.530 (2)	N6—C7	1.319 (2)
C2—H2A	0.99	N6—H6	0.88 (3)
C2—H2B	0.99	C7—N8	1.331 (2)
C3—C4	1.492 (2)	C7—H7	0.95
C3—H3A	0.99	N8—H8	0.84 (3)
C3—H3B	0.99		
			
C2—N1—H1A	113.7 (15)	H3A—C3—H3B	107.9
C2—N1—H1B	113.9 (15)	C5—C4—N8	106.22 (14)
H1A—N1—H1B	105 (2)	C5—C4—C3	132.25 (16)
C2—N1—H1C	112.9 (18)	N8—C4—C3	121.30 (14)
H1A—N1—H1C	103 (2)	C4—C5—N6	107.03 (15)
H1B—N1—H1C	108 (2)	C4—C5—H5	126.5
N1—C2—C3	109.22 (15)	N6—C5—H5	126.5
N1—C2—H2A	109.8	C7—N6—C5	109.26 (15)
C3—C2—H2A	109.8	C7—N6—H6	126.0 (18)
N1—C2—H2B	109.8	C5—N6—H6	124.3 (17)
C3—C2—H2B	109.8	N6—C7—N8	108.21 (15)
H2A—C2—H2B	108.3	N6—C7—H7	125.9
C4—C3—C2	111.78 (14)	N8—C7—H7	125.9
C4—C3—H3A	109.3	C7—N8—C4	109.27 (14)
C2—C3—H3A	109.3	C7—N8—H8	126.6 (18)
C4—C3—H3B	109.3	C4—N8—H8	124.2 (18)
C2—C3—H3B	109.3		
			
N1—C2—C3—C4	−170.28 (15)	C4—C5—N6—C7	0.6 (2)
C2—C3—C4—C5	26.5 (3)	C5—N6—C7—N8	−0.2 (2)
C2—C3—C4—N8	−159.79 (15)	N6—C7—N8—C4	−0.27 (19)
N8—C4—C5—N6	−0.70 (17)	C5—C4—N8—C7	0.61 (17)
C3—C4—C5—N6	173.73 (18)	C3—C4—N8—C7	−174.57 (16)

### Hydrogen-bond geometry (Å, º)

**Table:**

*D*—H···*A*	*D*—H	H···*A*	*D*···*A*	*D*—H···*A*
N1—H1*A*···Cl1^i^	0.92 (3)	2.19 (3)	3.1071 (17)	171 (2)
N1—H1*B*···Cl1	0.95 (3)	2.34 (3)	3.1930 (17)	150 (2)
N1—H1*C*···Cl2^i^	0.89 (3)	2.44 (3)	3.1768 (16)	141 (2)
N6—H6···Cl2^ii^	0.88 (3)	2.28 (3)	3.1046 (16)	157 (2)
N8—H8···Cl2^iii^	0.84 (3)	2.28 (3)	3.1157 (15)	169 (3)

Symmetry codes: (i) *x*+1, *y*, *z*; (ii) −*x*+2, *y*−1/2, −*z*+1; (iii) *x*, *y*, *z*+1.
