# Supplementary figures and images for: Crystal structure of 2-(1H-imidazol-3-ium-4-yl)ethanaminium dichloride, a re-determination
Source: Acta Crystallogr E Crystallogr Commun. 2015 Oct 14;71(Pt 11):o844–5. doi: 10.1107/S2056989015018848 (PMC4645084; doi:10.1107/S2056989015018848)

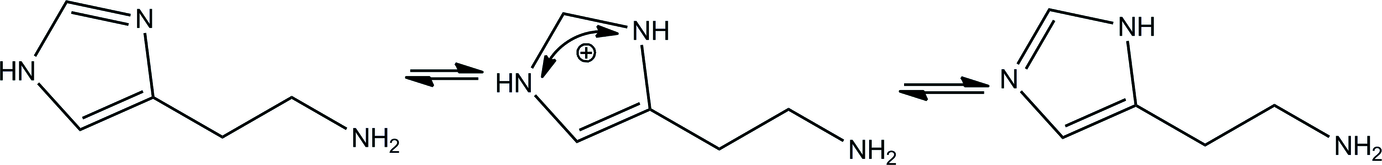

Supplement: Supplementary file 4 [file e-71-0o844-fig1.tif]

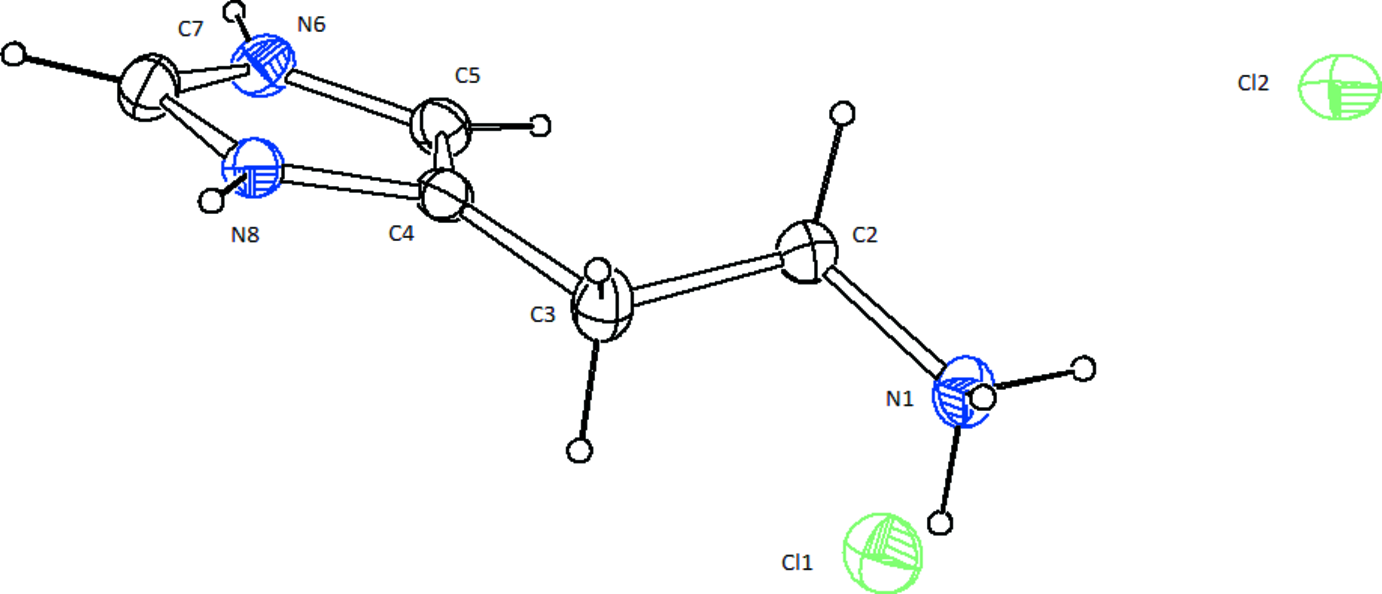

Supplement: Supplementary file 5 [file e-71-0o844-fig2.tif]

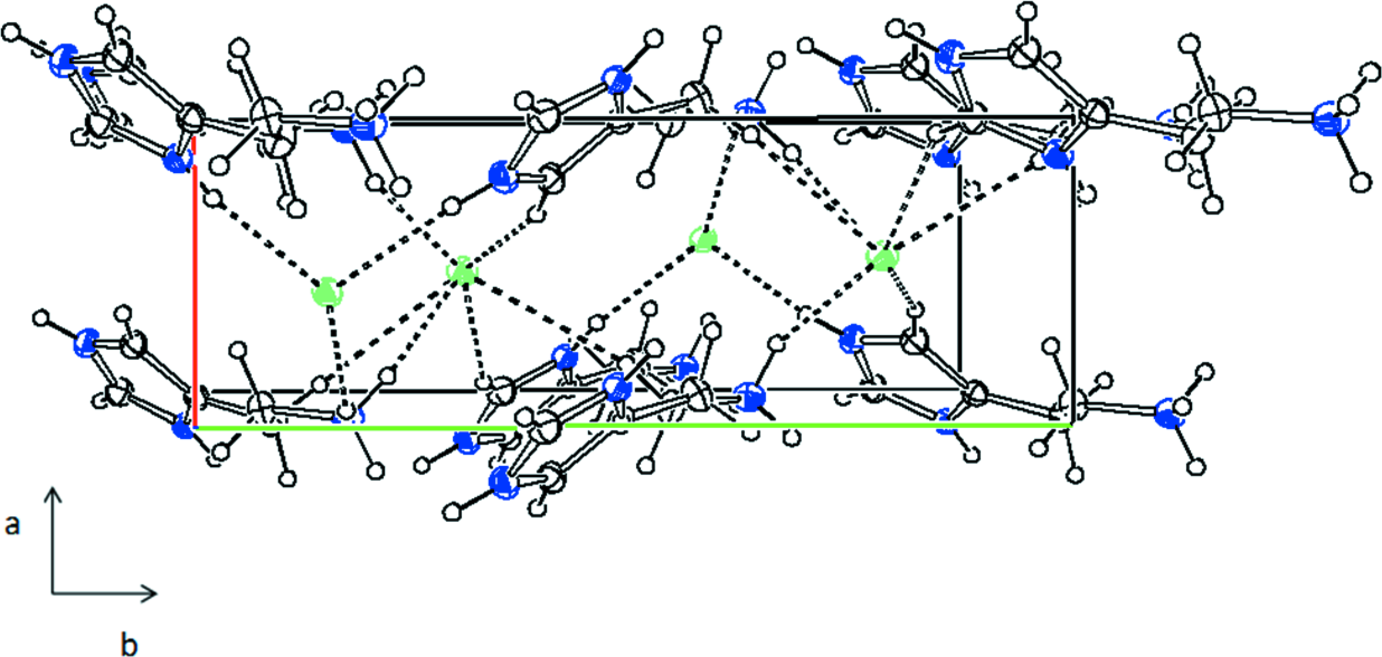

Supplement: Supplementary file 6 [file e-71-0o844-fig3.tif]
